# Severe fetal acidemia in cases of clinical chorioamnionitis in which the infant later developed cerebral palsy

**DOI:** 10.1186/s12884-015-0553-9

**Published:** 2015-05-27

**Authors:** Yoshio Matsuda, Masaki Ogawa, Akihito Nakai, Miki Tagawa, Michitaka Ohwada, Tsuyomu Ikenoue

**Affiliations:** Department of Obstetrics and Gynecology, International University of Health and Welfare Hospital, 537-3 Iguchi, Nasushiobara, 329-2763 Tochigi Japan; Department of Obstetrics and Gynecology, Tokyo Women’s Medical University, Kawada-cho, 8-1, Shinjuku-ku, 162-8666 Tokyo Japan; Department of Obstetrics and Gynecology, Tama-Nagayama Hospital, Nippon Medical School 1-7-1 Nagayama, Tama-City, 206-8512 Tokyo Japan; Department of Obstetrics and Gynecology, Miyazaki Medical Association Hospital, 738-1, Funado, Shinbeppuchou, Miyazaki-city, 880-0834 Japan

**Keywords:** Abnormal FHR pattern, Acidemia, Cerebral palsy, Clinical chorioamnionitis, Umbilical arterial pH

## Abstract

**Background:**

The umbilical arterial pH (UApH) in cases of clinically apparent chorioamnionitis (CAM) in which the infant later develop severe cerebral palsy (CP) has not yet been fully investigated. The objective of this study was to determine the UApH in CAM cases in which the infant later develop severe CP.

**Methods:**

A review was conducted unti1 April 2014 among 324 infants with CP diagnosed to be caused by antenatal and/or intrapartum conditions, as determined by the Japan Council for Quality Health Care. Eighty-six infants born at over 34 weeks of gestation with an abnormal FHR pattern during labor were selected. The subjects were divided into the following two groups: cases with (Group I, *n* = 19) and those without (Group II, *n* = 67) clinical CAM. Severe fetal acidemia was defined as a pH of less than 7.0.

**Results:**

The frequency of severe acidemia in Groups 1 and II was 26.3 and 74.6 %, respectively. In addition, the frequency of severe acidemia was significantly less in Group I (odds ratio (OR) 0.12, 95 % confidence interval (CI) 0.03–0.53) than in Group II, while the frequency of fetal tachycardia was greater in Group I (OR 7.61, 95 % CI 1.82–31.7) than in Group II, after adjusting for confounding effects.

**Conclusions:**

The frequency of severe acidemia was lower in the cases of clinical CAM in which the infant later developed severe cerebral palsy than in the cases without clinical CAM. The relation of fetal tachycardia to CP with clinical CAM, but not to acidemia, should be reevaluated in such cases.

## Background

Although birth asphyxia may cause cerebral palsy (CP), it has been demonstrated in controlled population-based studies that birth asphyxia does not account for most cases of CP [1]. It has also been reported that there is a close relationship between CP and clinically apparent chorioamnionitis (CAM) [2, 3], and CP has been shown to be induced by the direct effects of cytokines on the brain, even under conditions of mild hypoxic stress [3]. However, the relationships between infection/inflammation and the onset of CP remain unclear.

Severe/pathological fetal acidemia is defined as an umbilical arterial pH (UApH) of less than 7.0 and base deficit ≧12 mmol/l [4]. This condition is an objective measurement of the level of intrapartum hypoxia-ischemia and correlates with the occurrence of hypoxic ischemic encephalopathy [4]. The Japan obstetric compensation system for CP was established by the Japan Council for Quality Health Care (JCQHC) to compensate for CP resulting from intrapartum events and improve perinatal care. Using this system, we recently reported that placental abruption is the factor most associated with a low UApH and that even among infants with severe CP, over 10 % of all patients exhibited a non-acidemic status at birth [5]. In addition, there is a hierarchy, in which the lowest UApH is associated with placental abruption followed by an abnormal FHR pattern and an abnormal FHR pattern with CAM [5].

The present study was therefore performed to determine the UApH in clinically apparent CAM cases in which the infant later develop severe CP using the above compensation system for CP.

## Methods

This study was approved by the Ethics Committee of the International University of Health and Welfare Hospital, Tochigi, Japan.

The Japan obstetric compensation system for CP was launched on January l, 2009 to provide rapid monetary compensation for cases of CP [6] certified as first- or second-degree in severity according to the criteria of the Japanese Social Welfare System [7] in infants with a birth weight of over 2000 g and/or pregnancy length of over 33 weeks. In each case, a professional committee of the JCQHC consisting of physicians, midwives, lawyers and citizens carefully investigates the causative factors for CP and publishes a brief summary of the case with the cause of CP, with privacy protection, on their website [5, 8, 9].

A review was conducted unti1 April 2014 among 324 infants with CP diagnosed to be caused by antenatal and/or intrapartum conditions as determined by the JCQHC. A11 324 infants were born during or after January 2009.

Clinical chorioamnionitis (CAM) was defined as a maternal temperature of ≧ 38 °C and at least one of the following four criteria: maternal tachycardia of ≧ 100 bpm, uterine tenderness, white blood cell count of ≧ 15,000/mm^3^ or foul smelling vaginal discharge [10].

The FHR pattern observed two hours before delivery was defined as abnormal if one of the following patterns was detected: recurrent late decelerations, minimal or absent variability lasting for 40–60 min, severe variable decelerations, prolonged decelerations, tachycardia or bradycardia [11, 12]. Severe variable deceleration was defined as the lowest point, which was less than 70 bpm, and it continued for more than 30 sec, or the lowest point, which was more than 70 bpm and less than 80 bpm, and it continued for more than 60 sec [13]. These patterns were also used to assess the preterm fetuses. Bradycardia was defined as a baseline FHR of less than 100 bpm (>3 min) [12].

The causative factors for CP in 324 cases were classified according to the report by MacLennan (International Consensus Criteria) with modifications [12–15]. In 38 cases, the timing of the factor was thought to be an antenatal. Sentinel hypoxic event occurring immediately before or during labor were as follows: placental abruption (*n* = 87), cord prolapse (*n* = 16), a ruptured uterus (*n* = 15), maternal hypoxic events including amniotic fluid embolism (*n* = 8), and fetal exsanguinations due to vasa previa or feto-maternal hemorrhage (*n* = 8). Other factor suggesting that CP was caused by an event other than acute intrapartum hypoxia (perinatal hypoxia) was central nervous system or systemic infection except for clinical CAM (*n* = 8). The miscellaneous cases were 58 cases, including suspected cases which did not fulfil the criteria of clinical CAM (54 cases) or unknown cause (4 cases). The remaining 86 cases were divided into two groups: cases with (Group I, *n* = 19) and cases without (Group II, *n* = 67) clinical CAM, in order to clarify the characteristics of the cases of clinical CAM in which the infant later developed CP (Table 1).

**Table 1:** Causative factors in this study subjects

Causative factors	Numbers
Antenatal	38 (11.7 %)
Intrapartum	220 (67.9 %)
Placental abruption	87
Cord prolapse	16
Uterine rupture	15
Maternal hypoxic event	8
Fetal exsanguination	8
Clinical CAM (Group I )	19
Non clinical CAM (Group II)	67
Perinatal	8 (2.5 %)
Fetal central nervous system or systemic infection	8
Miscellaneous	58 (17.9 %)
Suspected CAM	54
Unknown causes	4
Total	324

Abbreviations, CAM: chorioamnionitis, suspected CAM: suspected cases which did not fulfil the criteria of clinical CAM

Severe/pathological fetal acidemia was defined as UApH of less than 7.0 and base deficit ≧12 mmol/l [4]. The distribution of UApH was graded as follows: less than 7.0 (severe/pathological acidemia) and more than 7.00.

The results are expressed as the mean ± standard deviation (SD), range (minimum to maximum value) or frequency (percent). The statistical analysis was conducted using the chi-square test, Fisher’s exact probability test or Mann-Whitney test, as appropriate. *P* values of less than 0.05 were considered to be significant. The odds ratios (ORs), adjusted ORs, and 95 % confidence intervals (CIs) were calculated to estimate the relative risk between Groups I and II. The variables were compared in both univariate and multivariate analyses. Logistic regression models were used to assess confounding effects. Our selection of the risk factors for inclusion in the regression model was based on the clinically important factors as well as the results of a univariate analysis.

## Results

### Clinical background factors in Group I

The characteristics of the 86 selected cases are summarized as follows: gestational age = 39.0 ± 1.5 weeks, birth weight = 2918.7 ± 499.1 g and UApH = 6.88 ± 0.18.

Table 2 shows the clinical characteristics of Group I. Rupture of the membranes before the onset of labor was seen in 11 cases (57.9 %), antibiotics were administered in 15 cases (78.9 %) and augmentation was performed in nine cases (47.4 %). The results of the detailed analyses of the FHR patterns are reported in Tables 3 and 4.

**Table 2:** Clinical characteristics of Group I (with Chorioamnionitis)

No	GW	BW [g]	ROM before labor	Mode of delivery	AS 1′/5′	UmApH/ base deficit	Inter-vention	Nonreassuring FHR pattern				
							Antibio/ Augment	PD	Mi/ AbVb	RLD	SVD	Tachy
1	35	2750	+	V	4/6	7.201/9.0	+/−	−	−	−	+	−
2	36	2552	+	V	0/0	6.876/23	+/−	−	+	−	+	+
3	38	2336	+	C	2/2	7.113/14.5	+/+	+	−	−	−	−
4	38	2386	−	V	1/3	7.104/9.2	+/−	+	−	+	−	+
5	39	2158	+	C	1/2	7.166/12.6	+/+	+	−	−	−	−
6	39	2540	+	I	1/1	7.016/13	+/+	+	−	−	−	−
7	39	2835	+	I	2/2	7.090/NA	+/−	−	−	−	−	+
8	39	2870	+	I	5/6	7.350/6.8	+/+	+	−	−	−	−
9	39	2980	−	C	1/4	6.943/17.7	+/−	−	−	+	−	+
10	39	3284	−	V	3/5	7.164/15.6	−/−	−	−	−	−	+
11	39	3364	−	C	1/5	7.080/19	+/−	+	−	+	−	+
12	39	3450	+	V	1/2	6.819/22	−/−	−	−	+	−	−
13	39	3860	+	V	1/4	7.191/11	−/+	+	+	−	−	+
14	40	3015	−	I	3/6	6.837/24	+/−	+	−	+	−	+
15	40	3048	+	C	1/3	6.849/21.1	+/−	+	−	−	−	−
16	40	3282	−	V	1/3	7.100/16	+/+	+	+	−	−	−
17	40	3436	+	I	3/5	7.194/6	+/+	+	+	+	−	+
18	41	2743	−	V	6/6	7.1379.6	−/+	−	−	+	−	+
19	41	3110	−	C	1/4	7.067/NA	+/+	+	−	−	−	+

Abbreviations, GW: gestational weeks at delivery, BW: birth weight, AS1′/5′: Apgar score at 1 /5 min, UmApH: blood pH of umbilical artery, ROM: from rupture of membranes Antibio/Augment: antibiotics or augmentation, FHR: fetal heart rate, PD: prolonged deceleration, Mi/AbVb: minimal or absent viability, RLD: recurrent late deceleration, SVD: severe variable deceleration, Tachy: tachycardia, V: vaginal, I: instrumental, C: cesarean, +: present, −: absent

**Table 3:** Comparison of the clinical characteristics of the two groups

	Group 1 (with CAM)	Group 2 (without CAM)	
	*n* = 19	*n* = 67	*p*
Gestational age at delivery	38.9 ± 1.5	39.0 ± 1.5	0.801
Birth weight (g)	2947.3 ± 440.9	2910.6 ± 517.2	0.759
pH less than 7.0 (severe acidemia)	5 (26.3 %)	50 (74.6 %)	<0.0001
Base deficit ≧12 mmol/l	11 (11/17, 64.7 %)	57 (57/62, 91.9 %)	0.018
Fetal growth restriction	4 (21.1 %)	14 (20.9 %)	0.76
Instrumental vaginal delivery	5 (26.3 %)	30 (44.8 %)	0.236
Cesarean section	6 (31.6 %)	18 (26.9 %)	0.890
Prolonged deceleration	12 (63.2 %)	58 (86.6 %)	0.048
Minimal or absent variability	4 (21.1 %)	30 (44.8 %)	0.104
Recurrent late deceleration	7 (36.8 %)	29 (43.3 %)	0.818
Severe variable deceleration	2 (10.5 %)	23 (34.3 %)	0.0920
Tachycardia	11 (57.9 %)	10 (14.9 %)	<0.0001

Abbreviations, CAM: chorioamnionitis

**Table 4:** Results of the univariate and multivariate analysis

	Univariate analysis	Multivariate analysis
Potential factors	OR (95 % CI)	Adjusted OR (95 % CI)
pH less than 7.0 (severe acidemia)	0.12 (0.04–0.39)	0.12 (0.03–0.53)
Base deficit ≧12 mmol/l	0.70 (0.49–0.99)	0.60 (0.09–3.63)
Prolonged deceleration	0.27 (0.08–0.85)	0.23 (0.04–1.39)
Severe variable deceleration	0.24 (0.05–1.13)	0.15 (0.02–1.09)
Tachycardia	7.84 (2.53–24.3)	7.61 (1.82–31.7)

Abbreviations, OR: odds ratio, CI: confidence interval

### Comparison of the clinical characteristics of the two groups and frequency of an abnormal FHR pattern in the two groups

Table 3 shows a comparison of the clinical characteristics of the two groups. According to the univariate analysis, the frequency of severe acidemia (Group I, 26.3 % and Group II, 74.6 %) and fetal tachycardia (Group I, 57.9 % and Group II, 14.9 %) was significantly different between the two groups. There were no significant differences between the two groups in terms of the other factors.

For factors considered to be possible factors, a further analysis was performed using a logistic regression analysis (Table 4). The results showed that the frequency of severe acidemia was significantly lower in Group I (OR 0.12, 95 % CI 0.03–0.53) than in Group II, while the frequency of tachycardia was greater in Group I (OR 7.61, 95 % CI 1.82–31.7) than in Group II, after adjusting for confounding effects.

## Discussion

The present study investigated the UApH values in cases of clinically apparent CAM in which the infant later developed severe CP. Consequently, the frequency of severe fetal acidemia was lower in these cases than in those without CAM. In addition, tachycardia was more frequent in the cases of clinical CAM, whereas the frequency of other abnormal FHR patterns did not differ statistically between the two groups.

Several mechanisms have been proposed to explain the association between clinical CAM and CP, including an inflammatory cytokine response, placental inflammation leading to impaired placental perfusion and gas exchange and intrauterine infection with bacteremia resulting in the direct involvement of the brain or fever associated with the infection itself exacerbating the primary insult to the brain [16]. Whether the presence of infection/inflammation, such as that associated with CAM, confers an additional risk for CP in term infants delivered in the presence of fetal acidosis has not been thoroughly studied. A single severe exposure, such as uterine rupture or massive abruption, may be sufficient to cause CP; however, much more often, it is not a single cause, but rather multiple concurrent risk factors, that precede the onset of CP. In the present study, the frequency of severe acidemia was significantly lower among the cases of clinical CAM with an abnormal FHR pattern than in those without clinical CAM. This finding indicates that brain injury may occur following a low level of hypoxic stress in the presence of clinical CAM. Garnier et al. [17] showed that intrauterine exposure to infection severely altered fetal cardiovascular function, resulting in dysregulation of cerebral blood flow and subsequent hypoxic-ischemic brain injury. In chronically instrumented fetal sheep, intravenous injection of lipopolysaccharide (LPS) decreased placental blood flow, whereas blood flow to the peripheral organ increased. The decrease in placental blood flow was accompanied by sustained hypotension, hypoxemia, and mixed acidosis causing dysregulation of cerebral blood flow. Because the simultaneous the change of FHR pattern was not examined in detail, further study is needed to show the relationship between the change of FHR pattern and the degree of infection.

In this study, we were unable to clarify characteristic FHR patterns associated with clinical CAM in which the infant later developed CP, as discussed by other researchers [18]. For example, it has been postulated that increased cytokines in such cases induce ischemia of the umbilical or uterine vessels, subsequently resulting in variable or late decelerations, in which the fetus may become hypoxic and acidotic.

In isolation, tachycardia is poorly predictive of fetal hypoxemia or acidemia, unless accompanied by minimal or absent FHR variability, recurrent late/variable decelerations or both [11]. On the other hand, Sameshima et al. [19] reported that the development of CP in pregnancies with intrauterine bacterial infection is associated with tachycardia, but not FHR deceleration patterns or acidemia, suggesting the presence of a different pathophysiology in these cases from that observed in the setting of acute hypoxia-ischemia.

In Japan, standardized FHR pattern management has recently been implemented [13]. Importantly, it has been reported that the degree of level has been ranked up when there are the presence of background complications, such as placental abnormalities or intrauterine fetal growth restriction. Clinical CAM should be included as such an obstetrical complication.

Our data are not derived from a nationwide population-based cohort, as an essential information was masked for personal protection. Nevertheless, the peer review system in Japan is the best clinically available method. It may be argued that our results are influenced by bias due to the small sample size; however, we consider this to be unlikely given the low-frequency nature of the cohort.

Further studies are needed to confirm the relationship between an abnormal intrapartum FHR pattern and the presence of clinical CAM by collecting data in such cases prospectively, including those in which the infant later develops CP.

## Conclusions

The frequency of severe acidemia was lower in the cases of clinical CAM in which the infant later developed severe cerebral palsy than in the cases without clinical CAM. The relation of fetal tachycardia to CP with clinical CAM, but not to acidemia, should be reevaluated in such cases.
